# Medical Textiles as Vascular Implants and Their Success to Mimic Natural Arteries

**DOI:** 10.3390/jfb6030500

**Published:** 2015-06-30

**Authors:** Charanpreet Singh, Cynthia S. Wong, Xungai Wang

**Affiliations:** 1 Australian Future Fibres Research and Innovation Centre, Institute for Frontier Materials, Deakin University, Geelong, VIC 3216, Australia; E-Mails: c.singh@deakin.edu.au (C.S.); cynthia.wong@deakin.edu.au (C.S.W.); 2 School of Textile Science and Engineering, Wuhan Textile University, Wuhan 430073, China

**Keywords:** vascular stent, graft, artery, weaving, knitting, electrospinning, braiding, compliance, non-linearity, anisotropy

## Abstract

Vascular implants belong to a specialised class of medical textiles. The basic purpose of a vascular implant (graft and stent) is to act as an artificial conduit or substitute for a diseased artery. However, the long-term healing function depends on its ability to mimic the mechanical and biological behaviour of the artery. This requires a thorough understanding of the structure and function of an artery, which can then be translated into a synthetic structure based on the capabilities of the manufacturing method utilised. Common textile manufacturing techniques, such as weaving, knitting, braiding, and electrospinning, are frequently used to design vascular implants for research and commercial purposes for the past decades. However, the ability to match attributes of a vascular substitute to those of a native artery still remains a challenge. The synthetic implants have been found to cause disturbance in biological, biomechanical, and hemodynamic parameters at the implant site, which has been widely attributed to their structural design. In this work, we reviewed the design aspect of textile vascular implants and compared them to the structure of a natural artery as a basis for assessing the level of success as an implant. The outcome of this work is expected to encourage future design strategies for developing improved long lasting vascular implants.

## 1. Introduction

The use of textiles for medical applications can be traced back to the early ages in wound care applications such as sutures and wound dressings. The suitability of textiles, whether in fibre or fabric form, lies in their structural flexibility, whereby some exhibit properties similar to human tissues, which are also composed of fibrous components. Another advantage of textile-based substrates is their design flexibility (from fibre to fabric stage), which can be modified to emulate the mechanical behaviour (elasticity, strength, stiffness, fluid permeability) of native biological tissue. These properties give textiles an edge over other materials (metals and plastics) in the area of soft tissue repair, for example, cardiovascular implants, which are used to replace/repair diseased arteries.

**Figure 1:**
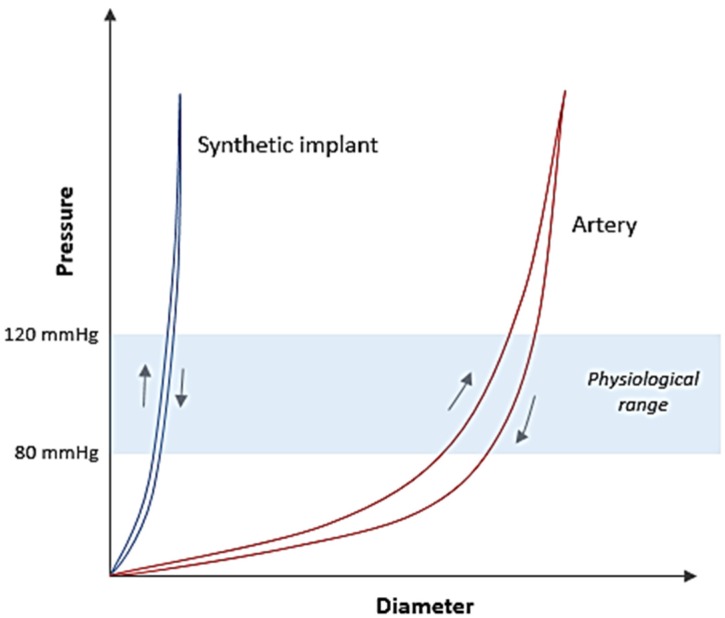
Comparison of pressure-diameter curves between an artery and a synthetic implant.

Cardiovascular implant market (grafts and stents) is growing at a fast pace due to increasing number of patients with vascular diseases and limited biological replacement options available. Thus, synthetic implants offer off-the-shelf solution in a range of design specifications. During the last two decades, a significant amount of research and industry effort has been put into developing vascular implants intended for various anatomical locations. Among the soft tissue implants, design optimisation of vascular implants is considered as one of the most complex tasks and has therefore been a continuing challenge for biomedical device engineers. The reason for this is the inability to match biomechanical behaviour of a synthetic implant to that of an artery. This is due to the heterogeneous structure of an artery, which imparts unique mechanical features (non-linearity, anisotropy, viscoelasticity, compliance) in the vascular wall. An artery shows very low stress response at low pressures and exhibits a steep increase in elastic modulus as the pressure is increased, a property known as non-linearity or incremental elastic modulus (Figure 1). Simultaneously, geometrical arrangement of structural components in the vessel wall imparts anisotropy characteristic to its mechanical behaviour. On the other hand, a synthetic arterial substitute (woven Dacron^®^ graft) is a homogenous structure, which exhibits low elasticity and relatively higher linear stress response at similar levels of strain (Figure 1) [1]. The viscoelasticity property is another important component that determines the hemodynamic behaviour of an arterial vessel and is an equally difficult feature to mimic in a synthetic implant. When an artery is subjected to cyclic inflation-deflation stresses, it does not react instantaneously to these stresses [2,3]. Instead, the artery, due to it being viscoelastic, produces a delayed response known as hysteresis when subjected to changes in pressure and volume (Figure 1). In addition to the biomechanical properties mentioned above, compliance, defined as radial extensibility of an artery in the physiological pressure range (80–120 mmHg) plays a decisive role in vascular mechanics. In a synthetic implant, this property is directly related to structural construction and material property, and hence highly variable. An unmatched compliance between an artery and implant is a common cause of long-term complications which result in ultimate failure of the surgical procedure due to altered pressure and flow dynamics [4,5]. Therefore, the design feature of medical textiles is critical when they are being considered for vascular implant applications.

Synthetic vascular implants are currently manufactured using standard textile manufacturing techniques such as weaving, knitting, braiding and electrospinning. A number of reviews have reported on the mechanical property comparisons of different types of vascular grafts and their clinical performance [6,7,8]. In 1986, Pourdeyhimi and Wagner presented an extensive review focusing on structures of the synthetic grafts to explain the reported clinical observations of these grafts [9,10]. However, recent studies on grafts and stents rarely consider the effect manufacturing techniques have on the structure. This paper will discuss the structure of vascular implants produced by different textile manufacturing methods and analyse them with respect to the arterial wall structure. The analysis is done while following the development timeline of these manufacturing techniques in the vascular implant industry. This approach will provide an understanding of the degree of success, which has been achieved by textile-based implants in mimicking the mechanics of native arteries since their first clinical introduction. Some innovative design concepts, which have attempted to reduce the mechanical property mismatch between the implant and the host artery, are also discussed. These works are thought to encourage the design of longer lasting vascular implants in future.

## 2. Artery: Structure and Mechanical Behaviour 

**Figure 2:**
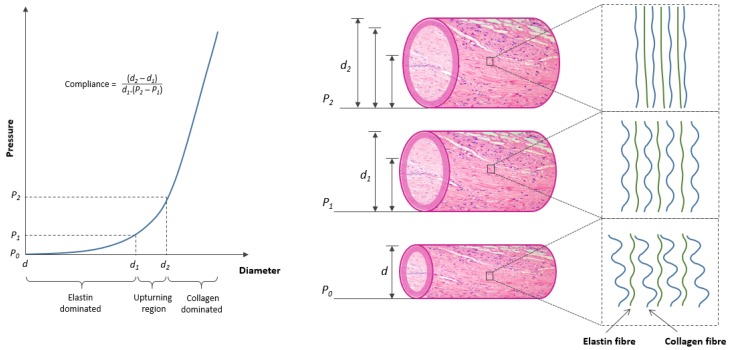
Role of fibrous components (elastin and collagen) in shaping the pressure-diameter relation of an artery.

The primary step towards designing a vascular implant with improved mechanical response is to understand the arterial structure, its components and mechanical role of each component. An arterial wall is composed of three major layers namely, tunica intima, tunica media, and tunica adventitia [11]. The intima or the endothelial layer comprises of a single layer of endothelial cells. The tunica media is the thickest layer and composed of circumferentially arranged elastic fibres, smooth muscle cells, and collagen fibres. The outermost adventitia layer comprises of large diameter collagen fibres oriented longitudinally as wavy bundles. The mechanical behaviour of an artery is based mainly on the thickness of media layer and its main structural components (elastin and collagen), which differ significantly in their elastic modulus (elastin = 0.6–1 MPa, collagen = 1 GPa) [2,3]. The function of both these components was investigated by Roach and Burton by selectively dissolving them and comparing the mechanical behaviour of the altered artery [1]. The study demonstrated that the initial slope in a typical stress-strain curve of an artery was contributed entirely by elastin fibres, while the final slope was due to collagen fibre stress response (Figure 2). The mid region (upturning region) involves successive transition or shifting of load from elastin to collagen and corresponds to the normal *in vivo* operating range (80–120 mmHg) of an artery. The non-linearity or incremental elastic modulus property is due to the wavy and random configuration of elastin and collagen fibres when unpressurised. With increase in pressure, elastin and collagen fibres start to straighten progressively. Elastin fibres become nearly straight at lower end of physiological pressure (80 mmHg). A further increase in pressure results in stretching of elastin and successive straightening as well as stretching of collagen fibres until top end of the physiological range (120 mmHg) is reached. An increase in pressure beyond this region (upturning region) results in fully stretched collagen and elastin fibres where stress response of collagen fibres dominates the arterial behaviour. This non-linear stress-strain behaviour is considered to be the key to elastic stability in arteries, which protects them from developing pathological conditions of aneurysms and ‘blowout’ at high pressures. The mechanical behaviour of arteries is also controlled by relative proportion of collagen and elastin fibres. High elastin content in ascending aorta (41% of dry weight) compared to descending aorta (30% of dry weight) is the reason behind a decreasing compliance trend observed while moving downstream in an aortic vessel [12,13].

**Figure 3:**
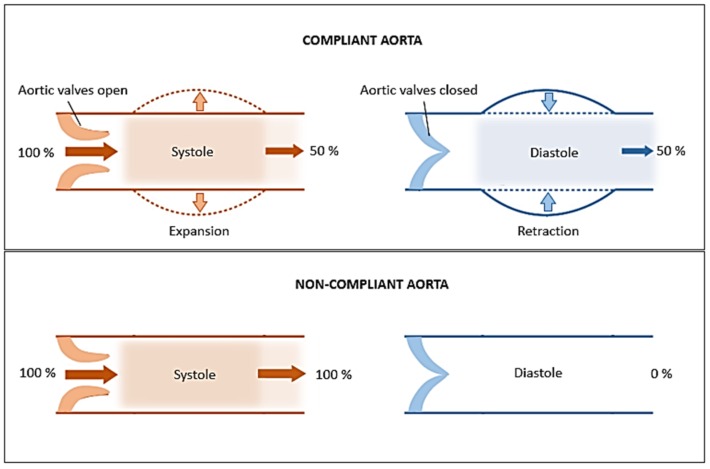
The role of compliance in the windkessel function of aorta.

The mechanical performance of an artery in the upturning region is expressed in terms of compliance value (percentage increase in diameter for a given increase in pressure) (Figure 2). A high compliance value indicates optimum level of structural performance for maintaining pulsatile blood flow. The property of an artery to support pulsatile flow is also known as the windkessel function, and is critically important in arteries in proximity of the heart such as the aorta [14]. During systole, the heart pumps nearly 60–100 mL of blood into the aorta while there is no supply during diastole. The windkessel function assists in temporarily storing a portion (approx. 50%) of systolic blood volume in aorta which can be later used during diastole to maintain consistent blood flow throughout the arterial network (Figure 3). During this process, the cross-sectional area of aorta (ascending aorta) can increase by 11% [15]. The basis of windkessel function lies in the compliance property, as a non-compliant (stiff) aortic vessel cannot expand sufficiently to store blood.

Anisotropy is another important characteristic of arterial wall attributed to circumferential orientation of collagen fibres and their unequal orthogonality in unstressed state. Arterial tissue is 40% stiffer in circumferential compared to axial direction and approximately 100% stiffer when inflated to physiological pressures [16]. Viscoelasticity or stress relaxation is a decisive component of arterial elasticity property. When an artery is subjected to cyclic inflation-deflation stresses, viscoelasticity causes hysteresis in pressure-volume curve. The area enclosed by hysteresis loop represents the energy lost in each cycle, which indicates that a major component of strain energy is recovered elastically each time arterial wall is distended [2]. The dissipation of strain energy due to viscoelasticity assists in attenuating forward pressure pulses, which propagate along arteries as waves of circumferential distention. Additionally, viscoelastic property has been suggested to improve the fatigue life of arteries by reducing dynamic stresses and strains in the wall [17].

## 3. Textile Structures as Vascular Implants 

The incorporation of textiles as vascular implants started in 1952 with the pioneering work of Voorhees and colleagues who replaced diseased aortic vessels of dogs with woven Vinyon-N (a polyvinylchloride) tubes [18,19]. Within few years of Voorhees’s work, a number of studies reported clinical trials with different types of materials (Nylon, Teflon^®^, Dacron^®^, Orlon^®^), and constructions (woven, knitted, braided) in various diameters (6–20 mm) [20,21]. The role of manufacturing technique should be considered when deciding on parameters such as the handling and *in vivo* behaviour of the grafts. The definition of an ideal vascular implant requires it to be (1) biocompatible, (2) non-thrombogenic, (3) compliant, (4) fatigue resistant, (5) flexible yet robust, (6) readily available, and (7) easy to manufacture. Among these, attributes 3–7 are influenced by the manufacturing method used and structural design of the implant, while attributes 1–2 are contributed by material selection. There are a number of techniques used to manufacture medical textiles and those that are used for vascular implants are mainly weaving, knitting, braiding and electrospinning. Each of these techniques presents specific characteristics and benefits which led to varying suitability for different types of vascular implants.

### 3.1. Weaving 

**Figure 4:**
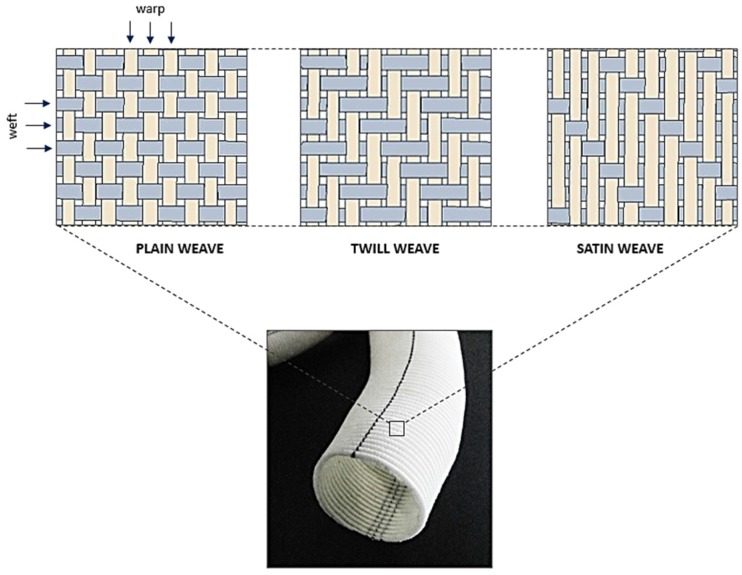
Structural design patterns of a woven Dacron^®^ graft.

A woven graft is manufactured by interlacing two sets of yarn (warp and weft) oriented at 90° to each other. These grafts are currently available in different types of weave designs namely, plain, twill, and satin (Figure 4). The main characteristics which were considered important while introducing these grafts in clinical practice were surface smoothness, handling ease, non-reactivity, water permeability, bursting strength, suture retention strength, and biological healing response [22,23,24,25,26]. On the other hand, biomechanical optimisation of graft design was largely overlooked in the last six decades since the conception of this great innovation. This is also indicated by the fact that the basic construction (a homogenous single layer woven fabric) of a woven graft has remained the same since its introduction in the 1950s.

A commercial woven graft can be considered similar to a windcheater jacket from structural and material perspective, and bears no relationship to the composite design principle of an artery. The dissimilarity is apparent when the fibre itself is analysed, whereby the elastic behaviour of polyester fibres corresponds to collagen fibres while no graft component performs the role of elastin fibre, *i.e.*, low stress elastic response (Table 1). Furthermore, at the structural level, the straight yarn interlacement in woven grafts is unlike the helically arranged wavy configuration of collagen fibres, which induces non-linearity property in an artery. The orthogonal arrangement of both yarn components results in low axial elongation and poor radial compliance properties in these grafts [27]. The desired axial elasticity is often achieved through graft crimping, which also helps maintain tubular shape of graft during bending. However, optimum compliance is still an unachieved goal even with numerous proofs of its clinical importance [28,29,30].

**Table 1:** Elastic modulus of components of an artery [31] and a woven Dacron^®^ graft. (* Stainless steel strut in a woven stent-graft).

Structure	Component	Elastic Modulus (MPa)
Artery	Elastin	0.6–1
	Collagen	1000
	Smooth Muscle	0.1
Woven graft	Dacron^®^ Polyester	800–900
	Stainless steel*	190 × 10^3^–210 × 10^3^

The consequences of poor compliance can be fatal as it can change the transmission characteristics of pulse waves [4,32,33]. Moreover, a compliant host artery expands more than the stiff graft, which creates an abnormal stretch on the suture line. The continuous stretch subsequently produces structural fatigue of the arterial wall at the anastomosis [5]. A significant amount of research has been done on optimising construction and handling properties of woven grafts [34,35,36,37,38,39] but radial compliance/elasticity is still an unresolved issue [27,40] and has a direct influence on long term patency of these grafts (Table 2).

**Table 2:** Comparison of compliance property of Dacron^®^ grafts with natural blood vessels. Adapted from [33].

Structure	Compliance (mmHg × 10^−2^)
Artery	7.4
Vein	2.7
Dacron^®^ Polyester (Woven)	1.9
Dacron^®^ Polyester (Knitted)	2.3

There are very few studies that report initiatives to match graft mechanics to the host artery. These include studies published soon after the work of Voorhees, which attempted to tackle graft plasticity by using elastic filaments (Lycra^®^ spandex) [41], and crimped synthetic filaments (Helanca^®^ nylon and Dacron^®^ polyester) [42,43]. Their concept was based on mimicking the role of wavy collagen and elastin fibres by using crimped and elastic yarns, respectively. Later, the combined use of both these components in plain weave constructions was successfully reported [44,45]. This approach improved the compliance nearly 17 times compared to a commercial woven graft (0.0324 ± 0.0083 kPa^−1^ *vs.* 0.00186 ± 0.0005 kPa^−1^) and the new compliance matched that of a human common carotid artery (0.0238 ± 0.0132 kPa^−1^) [44]. However, from the design perspective, woven graft was still in its primitive single layer fabric stage.

**Figure 5:**
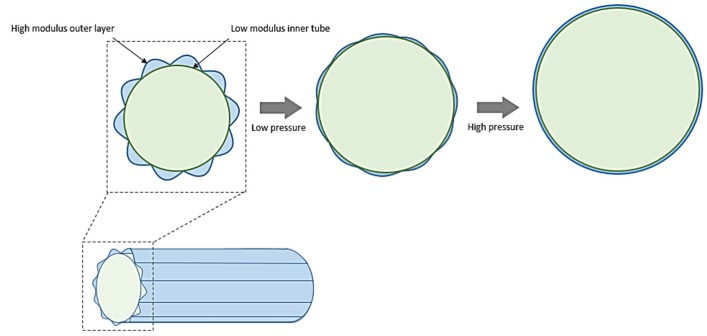
An explanation of thebilayer woven graft design concept proposed by Chen *et al.* [46].

In 2011, an innovative woven graft design concept was reported by Chen *et al.* based on the coaxial graft concept of Sonoda *et al.* [46,47]. In this work, a bilayer woven graft prototype was developed in which the inner layer was constructed from low modulus yarns (poly-trimethylene terephthalate) while the outer layer made from high modulus yarns (polyester) was stitched in a crimped form to the inner layer (Figure 5). This type of structure can mimic the layered structure of blood vessels in a way that the strain at lower pressures is absorbed by the inner layer while a further increase in pressure will cause the outer layer to uncrimp and “join-in” with the inner layer to increase the overall elastic modulus of the graft. The authors reported a similar observation where elastic modulus of the new graft remained low, up to 80 mmHg and increased rapidly afterwards [46]. However, a drawback of this design is reduced bending flexibility or kink resistance due to the crimped outer layer, which may limit its clinical applicability.

While *in vitro* studies can provide proof of concept confirmation for new designs, *in vivo* studies, whether on animals or human beings, provide more convincing evidence of their performance in actual clinical environment. One of the early long-term animal studies established the advantage of bi-component graft design (spandex and Dacron^®^) in improving graft mechanical properties [45]. The graft was sufficiently compliant (6.9%, 80/100 mmHg; 9.5%, 80/200 mmHg) even after one year of implantation and did not show any plastic dilatation at explantation after three years. Unfortunately, no published *in vivo* trial data is available for rest of the new designs discussed above [44,46,47].

### 3.2. Knitting

**Figure 6:**
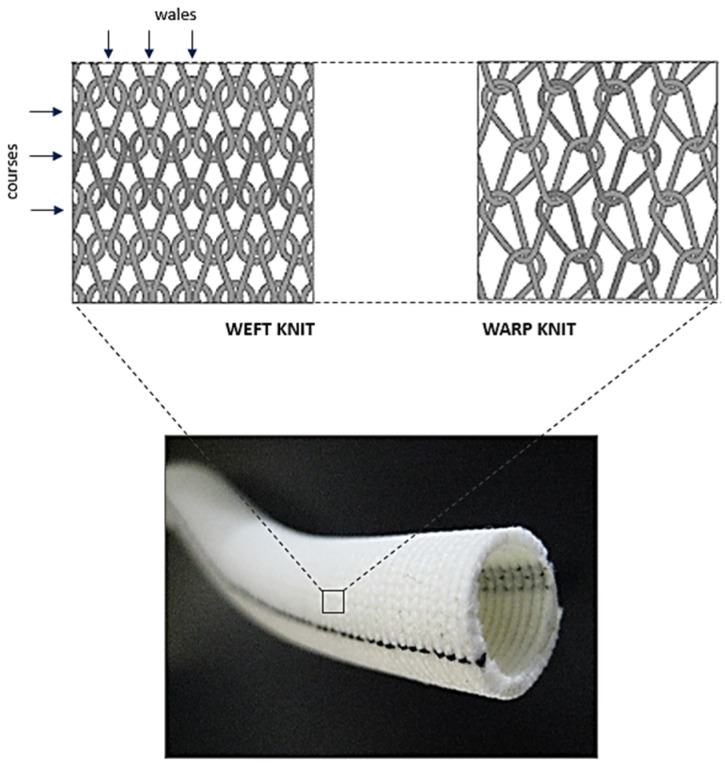
Structural design patterns of a knitted Dacron^®^ graft.

Knitted grafts have a looped filament construction in which a continuous interconnecting chain of yarn loops spirals around the graft circumference. Knitted structures are softer, more flexible, compliant, and have better handling characteristics than woven structures. The most common types of knits, which are used for graft design, are the weft knit and warp knit constructions (Figure 6). Warp knitted structures have less stretch than weft knits, and therefore are inherently more dimensionally stable. The knitted grafts were first introduced in clinical practice in 1955 with the intent to remove the seam problem in a woven graft [48]. Later, in 1958, the renowned heart surgeon Dr. Michael DeBakey firmly established the clinical usefulness of knitted Dacron^®^ grafts [49,50]. The trend of using Dacron^®^ as a standard graft material started thereafter owing to the better long-term biostability of Dacron^®^ compared to other available materials (Nylon and Orlon^®^). Improved stretch widthwise (radial compliance) compared to woven grafts was a promising observation in knitted grafts. However, high porosity and long-term dilatation were also reported [26,41]. The reason being absence of structural heterogeneity in knitted grafts as in arteries which protects them from undergoing fatigue dilation. Since then, several studies have been published reporting the use of different coating techniques, constituent materials, and knitted patterns with a motive to improve handling, fatigue and biological performance of these grafts [51,52,53,54,55,56,57,58,59]. Based on the inherent structural flexibility of knitted structures, many investigations have reported their use as elastic tubular substrates [60,61,62,63,64]. This property also formed the reason behind the use of monofilament knitted mesh structures as vascular stents [65,66,67,68,69]. However, commercially available knitted implants do not vary much in their structure, which is mainly a plain knit single layer homogenous structure bearing resemblance to an upholstery fabric rather than to an artery. In addition, Dacron^®^ has been the only material for commercial knitted grafts since its use was first proposed by DeBakey [49]. Although Dacron^®^ grafts are currently used in the clinics, there is a significant mismatch between their mechanical behaviour in comparison to natural arteries (Table 2) [40].

Similar to woven grafts, knitted grafts also underwent design trials to improve their elastic behaviour. The first report on use of spandex filament knitted graft as a dog abdominal aorta (diameter 8–10 mm) was presented by Wagner *et al.* [41]. However, the solo use of spandex fibre as graft material was observed to cause long-term dilatation defect in the graft and is attributed to homogenous single layer structure of the graft. Some of the latest studies tried to use a composite polyester/spandex filament yarn to improve graft elasticity [62,64]. This type of material composition allows load sharing among both components and can prevent dilatation issues if structural design pattern is also modified. However, these studies only report basic improvements in mechanical properties of a plain weft knit structure and lack the ability to be considered as a significant design improvement to mimic arterial mechanics in a knitted graft.

**Figure 7:**
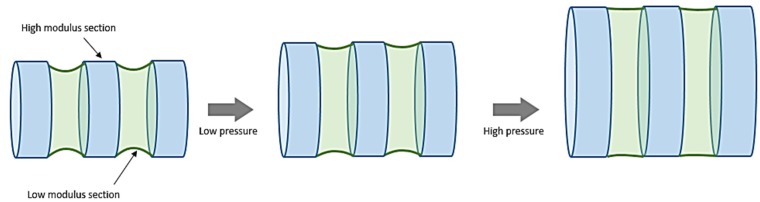
The segmented design concept as proposed by Singh and Wang to improve the compliance property of a knitted vascular implant [70,71].

In a recent study, an innovative knitted stent-graft design was reported which closely mimics the natural artery mechanical behaviour [70]. The design is based on the concept of longitudinal structural segmentation or metamerism in which the knitted tube is divided into multiple low and high modulus segments arranged in alternating sequence (Figure 7). The low modulus (knitted polyurethane) sections tend to remain contracted (reduced diameter) when unpressurised while high modulus (knitted polyester) maintain the as-knit configuration. Therefore, at low internal pressure, the expansion of low modulus segments controls the stress response of the knitted tube until their circumference equals that of high modulus segments. At high pressures, the combined response of both the segments increases the stress response sharply, exhibiting an incremental elastic modulus property similar to natural arteries. The low modulus segments act as intermittent “buffer zones” which assist in radial expansion as well as provide a kink-free configuration to the knitted tube. The compliance of this new design (volumetric: 0.056 ± 0.006 mL/mmHg; radial: 9.8 × 10^−4^ mmHg^−1^) is nearly 7 and 15 times better compared to a conventional knitted stent (radial: 1.45 × 10^−4^ mmHg^−1^) and a commercial woven Dacron® graft (volumetric: 0.0038 ± 0.002 mL/mmHg), respectively, and falls well within the physiological range of aortic vessel [70,71]. However, the *in vivo* performance of this design is still unavailable to demonstrate its clinical performance.

### 3.3. Braiding

The application of braided textiles in traditional application areas (apparel, upholstery) is very limited compared to knitted and woven structures and is mainly found in technical textiles (industrial, sports, automotive). Braiding technique involves the use of three or more component yarns, which are intertwined at an angle to each other. In other words, braided structures are similar to traditional woven structures but with an angle bias. The angled mesh structure allows easy radial expansion of braided tubes. This property formed the basis of using braiding in the early years of textile graft innovation [72,73,74,75].

The advent of endovascular technology in the 1990s led to the search for user-friendly stent designs [76]. The ability of tubular braided structures to compress easily and recoil back made them a promising candidate for self-expanding vascular stents [77]. Low bending rigidity of braided mesh tube was also an additional advantage for stent application. However, radial expansion in braided tubes occurred at the expense of axial shortening and elastic recovery was limited to open mesh braids only. Also, braided stents were not very successful in endograft application as it required suturing/bonding of a tubular graft to stent wires and, hence, limited the movement of constituent wires at their cross-over points which ultimately led to stent failure (wire breakage) under cyclic, *in vivo* conditions [78]. This limitation shifted braided stents towards stent applications which do not require graft covering, suitable for peripheral atherosclerotic arteries, which is also their current main application area (Table 3) [79].

**Figure 8:**
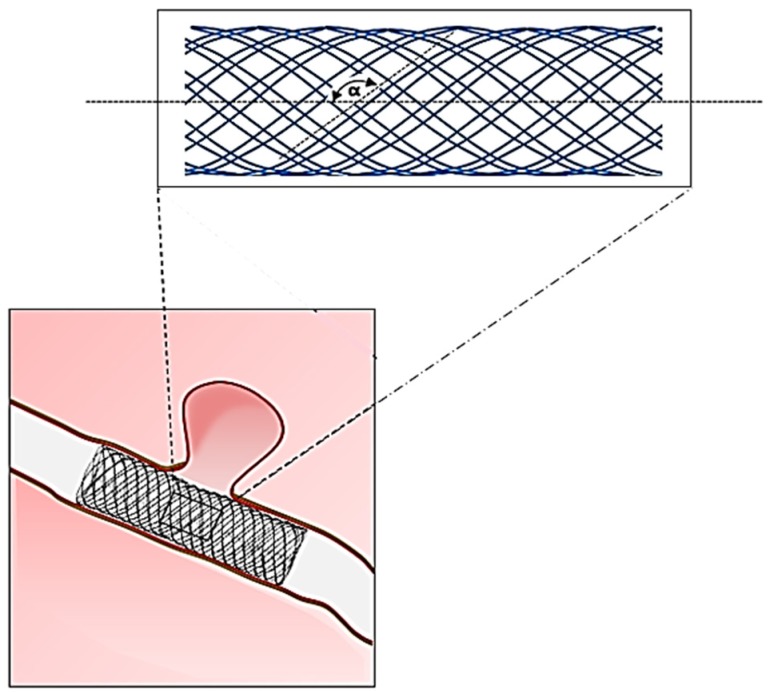
Structural geometry of a braided metallic stent (α = braid helix angle).

The commercially available braided stents are made from various metallic alloys (Table 3), which make them highly non-compliant and mechanically similar to a braided plumbing hose (Figure 8). Since low porosity is an important requirement in flow diverter stents, the number of wires is generally kept high (up to 96) to increase mesh density [80,81,82,83]. Similarly, compression of atherosclerotic region requires high radial compression strength, which is generally achieved by increasing mesh density. However, an inverse effect of high mesh density is increased stent stiffness (radial and longitudinal), which has a negative impact on the host artery hemodynamics [84,85]. The arterial length covered by the stent becomes non-compliant and results in pressure attenuation across this region and localised increase in pulse-wave velocity. A stiff metallic stent also causes straightening of the host artery and may induce kinks [86]. The development timeline of braided vascular stents to date mainly includes trials with various polymeric filaments [65,87,88,89,90,91,92,93,94,95,96] and stent parameter optimisation studies [89,96,97,98,99,100], while improvement in conventional braided design (a single component homogenous construction) to match arterial mechanics was rarely investigated.

**Table 3:** Categorisation of commercial braided stents according to their application area, material and design feature.

Trade Name	Manufacturer	Application	Material	Design Feature
PIPELINE^®^	ev3 Inc.	Flow diverter stent	Cobalt-Chromium + Platinum	Single layer braided tube
p64^®^	Phenox GmbH		Nitinol	
LEO^®^ PLUS	Balt Extrusion		Nitinol	
SILK^®^			Nitinol + Platinum	
LVIS Device^®^	MicroVention Inc.		Nitinol	
WALLSTENT™	Boston Scientific Co.	Carotid stent	Elgiloy^®^	
ROADSAVER^®^	Terumo		Nitinol	
SUPERA^®^	Abbott Vascular	Peripheral stent	Nitinol	
Agili-D^®^	Altura Medical	Abdominal endograft	Metal alloy	

The research contribution towards improving braided stent mechanics is very limited but still worth mentioning. The concept of using a multilayer braid design was introduced to reduce porosity of the stent while maintaining the bending flexibility, which otherwise becomes too low in a high mesh density single layer stent to be able to conform well to the arterial wall. The use of multiple layer braided stent has been trialled in many clinical studies and is commercially available as CARDIATIS^®^ multilayer stent [101,102,103]. Another new design concept was reported recently which is a single layer braided structure but with two different zones/sections along its length [104,105]. The central zone (70% of stent length) is a tightly braided high mesh density section while the margins have low mesh density. This allows reduced blood flow to the diseased portion of the blood vessel while maintaining flexibility in rest of the “non-functional” stent length. The commercial version of this design is available as E-volution^®^ stent (Jotec GmbH, Germany). Marchand *et al.* tried to improve the radial compliance of a braided heart valve stent by optimising shape-setting parameters of the stent material (nitinol alloy) [106]. The modified heat treatment process was reported to improve radial expandability of the stent when tested in an *in vitro* test experiment.

### 3.4. Electrospinning 

Since their introduction, Dacron^®^ grafts (woven and knitted) have dominated the vascular graft market in large diameter (15–30 mm) blood vessel replacement. Dacron^®^ grafts have also been used as replacements for small diameter (<6 mm) arteries (coronary, below-the-knee, tibial, and peroneal). However, there were a number of limitations identified such as owing to mismatched compliance, graft thrombosis, and anastomotic intimal hyperplasia (Table 4) [6,23,107,108,109,110]. One of the most commonly used strategies to address some of these limitations is by coating the synthetic grafts with protein such as collagen. As shown in Table 5, the general trend observed is that grafts coated with collagen, fibronectin or heparin demonstrated better cell attachment *in vitro* and improved patency *in vivo* [111,112,113,114].

**Table 4:** Compliance and cumulative patency of different arterial grafts implanted in a small diameter (femoropopliteal artery) position. Adapted from [115]; PTFE, Polytetrafluoroethylene.

Graft Type	Compliance (% mmHg × 10^−2^)	Structure	Patency % (30/180 days)	Patency % (1/2 years)
Human femoral artery	5.9	–	–	–
Saphenous vein	4.4	Natural tissue	94/93	88/84
Umbilical vein	3.7	Natural tissue	97/93	83/80
PTFE	1.6	Extruded (non-textile)	85/81	60/42
Dacron^®^	1.9	Woven	88/72	65/42

**Figure 9:**
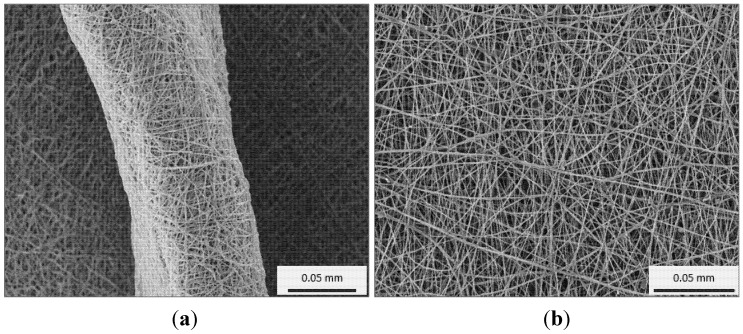
The wall thickness (**a**) and surface (**b**) view of an electrospun mesh.

The graft structure (porosity, fibre diameters, pore connectivity, surface area, and compliance) is important as it influences cell growth behaviour. This led to the development of a new textile fibre spinning technique known as electrospinning, which is capable of producing fibres to the scale of native collagen and elastin fibres. In an electrospinning process, a strong electric field is generated between a polymer solution (delivered through a syringe needle) and a metallic collector. When the voltage reaches a critical value, the charge overcomes the surface tension of the polymer solution drop at the needle tip and polymer jet is generated. While travelling towards the metallic collector the drawing force exerted by electric field and simultaneous evaporation of the solvent results in reduction in the diameter of the jet. The collected dry fibres form a nonwoven mesh of nanometre to micrometre diameter fibres (Figure 9). The process can be adjusted to control fibre diameter to some extent by varying the charge density and polymer solution concentration. The feature of producing very fine fibres increases the surface area significantly in electrospun meshes, which is a beneficial property for improving cell growth [116].

The structural construction of an electrospun fibre scaffold can closely mimic the natural extracellular matrix structure [117]. Electrospinning also enables the use of a wide range of natural and synthetic polymers [118], thus, increasing the possibility of matching both the biological and mechanical properties similar to an artery (Table 5) [119]. The electrospinning of natural proteins like collagen and elastin have demonstrated the usefulness of this technique to bring synthetic vascular grafts near to their biological counterparts [120,121]. From a structural design perspective also, electrospinning provides a range of possibilities in mimicking the multilayered construction of arterial wall [122,123,124,125]. The use of different polymeric components in individual layers provides more flexibility in controlling the graft mechanical response from each structural layer.

**Table 5:** Biological responses in various types of synthetic vascular grafts created from different manufacturing techniques.

Technique	Material	Scaffold Dimensions	Biological Response	Mechanical Testing	Ref.
Knitted	PET	4 mm diameter	ECs: Better cell attachment was observed on precoated grafts in the following order: collagen (3.5 × 10^5^ cells/cm^2^) > fibrin (2.8 × 10^5^ cells/cm^2^) > fibronectin (2.4 × 10^5^ cells/cm^2^) > laminin = untreated (1.3 × 10^5^ cells/cm^2^)	–	[126]
Knitted	PET	4 mm diameter cut into fusiform patches of 5 cm length × 8 mm width	*In vivo*: Implanted in sheep for 4 weeks and intimal hyperplasia assessed. Fluoropolymer coated PET sealed with gelatin produced the least amount of hyperplasia followed by PTFE, carbonlined PTFE and gelatin sealed PET	–	[127]
Knitted	PET	–	*In vivo*: Randomised clinical trial of 209 patients (femoropopliteal bypass). Patency at 3 years of collagen-coated heparin bonded PET (54%) was better than PTFE (44%). No difference in patency at 5 years between PET and PTFE	–	[128]
Woven and Electrospinning	PET and PU	Flat	*In vitro* (HUVECs): All 3 materials showed better cell attachment when coated with Collagen Type I/III as compared to their uncoated counterparts. Cell coverage on coated PTFE (34.6%) > PET (19.6%) > PU (17.5%); When exposed to shear stress of 1 Pa for 1 h, cell retention was highest on coated PTFE. No difference was observed across the 3 materials when shear stress increased to 2 Pa	–	[129]
Electrospinning	PCL and PU	NS	*In vitro* (HUVECs): Good cell attachment (~ 61%). Cells produced proteins such as PECAM and vWF, indicating normal phenotype and function	–	[130]
Electrospinning	Silk fibroin	1.5 mm diameter	*In vivo*: Implanted in rats for 7 days to 6 months. No graft degradation or foreign body reaction observed. 95% of grafts remained unblocked. After 1 month implantation, ECs were observed on the luminal surface of the grafts. Cell coverage continue to increase at 6 months	–	[131]
Electrospinning	Silk	5 mm diameter	*In vitro*: ECs and SMCs attached and proliferated on the scaffold. ECs were observed on the surface of the scaffold while SMCs migrated into the scaffold	Tensile strength: 2.42 MPa; Elastic modulus: 2.45 MPa; Mean burst pressure: 811 mmHg	[132]
Electrospinning	PCL	2 mm diameter	*In vivo*: Implanted in rats for 24 weeks. No narrowing of the grafts (stenosis) in the PCL group. ECs coverage confluent at 12 weeks in the PCL group *vs.* ePTFE group (incomplete at 24 weeks).	–	[133]
Electrospinning	PCL	NS	*In vitro*: A confluent layer of oriented SMCs in the direction of aligned fibres after 7 days culture	–	[134]
Electrospinning	Collagen Type 1, elastin and PLGA	Tubular (4.75 mm diameter, 12 cm long)	*In vitro*: Mean of 72% for ECs and 82% for SMCs viability over 7-day culture period; *In vivo* (mice): No systemic or neurological toxicity, normal blood count, transient local inflammation at implantation site	Burst pressure: 1425 mm Hg; Compliance: 12%–14% for scaffold *vs.* 9% for native vessel; Maximal axial and circumferential strain: 40% strain	[119]
Electrospinning	Collagen and PCL	Tubular (4.75 mm diameter, 12 cm long)	ECs: Cytoskeletal organisation and focal adhesion via actin and vinculin staining respectively were better developed when cultured on smaller sized fibres; SMCs: Infiltration of cells into scaffolds with fibres > 1 µm in diameter during a 4-week culture	Tensile strength: Increasing fibre diameter (0.27 µm to 4.45 µm) decreased tensile strength from 3.15 MPa to 0.75 MPa; Elongation at break: Increased with increasing fibre diameter (90% for 0.27 µm fibres to 734% for 4.45 µm fibres); Maximum load: Decreased from 25.75 N (0.27 µm fibres) to 8.63 N (4.45 µm fibres)	[135]
Knitting	Elastin solubilised proteins and PET	Flat	HUVECs: No cytotoxicity, 43% cell attachment for elastin solubilised protein-PET *vs.* 94% for PET	–	[136]
Electrospinning	Elastin and PDO	6 mm diameter	*In vitro*: Fibroblasts cultured for 7 days on PDO:Elastin graft showed migration into the fibrous materials *vs.* no migration in PDO graft	PDO:Elastin ratio of 50:50 produced compliance that mimics native femoral artery	[137]
Electrospinning	PCL, PDO and Silk	NS	*In vitro*: Risk of clotting using human monocytes – PCL < Silk < PDO. The risk in PCL is comparable to ePTFE (currently used grafts in clinics)	–	[138]
Electrospinning	Chitosan and PVA	NS	*In vitro*: Good fibroblast growth was observed with continual proliferation up to 21 days	–	[139]

EC = endothelial cell; ePTFE = expanded polytetrafluoroethylene; HUVEC = human umbilical vein endothelial cell; NS = not specified; PCL = polycaprolactone; PDO = polydioxanone; PET = polyethylene terephthalate; PLGA = poly(lactic-co-glycolic acid); PTFE = polytetrafluoroethylene; PU = polyurethane; PVA = polyvinyl alcohol; SMC = smooth muscle cell.

## 4. Discussion 

The woven graft development timeline suggests that this technology has shown biomechanical benefits of using new structural components but there are very limited attempts which focus on improvising conventional weave designs to suit the arterial site. Knitted structures appear to be a suitable candidate for vascular implant application owing to their inherent structural and design flexibility. Also, anisotropic elasticity property (axial > circumferential) in knitted structures is similar to that of the native artery, which is an advantage over woven structures. Currently, some of the latest developments in woven and knitted grafts use biological coatings to improved tissue growth and design modifications to match the implant site anatomy, while the lack of innovation from material and biomechanical aspect is evident in new commercial products (Table 6). The future products may require a combined input from advanced textile designing and biomechanics together in order to realise the full clinical potential of textile grafts. The helical arrangement of constituent filaments/wires give braided structures a design advantage over woven and knitted structures to mimic the helical geometry of collagen and elastin fibres in an artery. However, the focus on this aspect of braided stent improvement is mostly unrealised. Since, braided stents are deployed in their fully expanded state (helix angle approaching 90°), the low stress radial expansion property owing to helical geometry is completely lost (Figure 8). The use of elastic filaments and design of bio-component structures can be a future prospect for developing compliant braided stent devices. Expanded polytetrafluoroethylene (ePTFE) is an inert fluorocarbon polymer (stiffness: 0.5 GPa, tensile strength: 14–18 MPa), which is developed by heating, stretching, and extruding process resulting in a porous polymeric structure. Non-textile grafts made from ePTFE are currently used in the clinics as medium diameter grafts (7–9 mm) for peripheral vascular diseases. However, ePTFE grafts are not viable as conduits for small diameter (<6 mm) vessel replacement due to a high rate of occlusion. Although ePTFE is a chemically inert material, the structural stiffness and low radial compliance of these grafts contributes to their poor long-term patency (Table 4). There have been numerous investigations into endothelial cell seeding and surface functionalization of ePFTE grafts to improve their clinical performance as small diameter grafts. Current efforts have yet to produce a small diameter synthetic graft that is comparable to an autologous graft. Electrospinning technique has proven to be a promising option for small diameter grafts in many *in vitro* and animal studies. In comparison to established textile manufacturing methods (weaving, knitting, braiding), this technology is equipped with much higher levels of design flexibility in terms of material variety (natural, synthetic), structural heterogeneity (multi-layer, multi-component), and therapeutic ability (drug delivery). However, development of an off-the-shelf electrospun graft product capable of achieving rapid cell coverage with minimised risk of thrombosis, intimal hyperplasia, and mechanical failure has yet to be achieved.

Radial compliance is unarguably the most vital mechanical feature requirement from a synthetic vascular implant. In conjunction with compliance, non-linearity and anisotropy are two critical mechanical characteristics, which a vascular graft must also possess in order to successfully mimic natural artery mechanics. Non-linearity prevents the late development of pathological conditions related to graft failure at high pressures [2,3]. Anisotropic mechanical property prevents excessive stimulation of anastomotic region, while being compliant in radial direction to prevent any flow disturbances [125]. In an artery, both these properties are achieved owing to the multilayered heterogeneous structure of arterial wall, which a single-layer single-component isotropic textile structure is unable to mimic.

**Table 6:** A list of selected commercially available Dacron^®^ grafts and their design features.

Structure	Material	Trade Name	Special Design Feature	Intended Improvement	Manufa-cturer	Application Area
Woven	Dacron^®^ & PTFE	FUSION BIOLINE^®^	Two layer, Heparin coated	Improves patency and healing response	Macquet	Peripheral
	Dacron^®^	HEMASHIELD^®^ PLATINUM	Multiple branched, Double velour, Collagen coated	Enhances healing response		Aortic
		VASCUTEK^®^ GELWEAVE™ Pre-curved	Pre-curved design	Matches aortic arch anatomy	Terumo	
		VASCUTEK^®^ SIENA™	Extended trunk and collar design	Suits hybrid surgery procedures		
		VASCUTEK^®^ GELWEAVE™ Plexus	Multiple branched, Gelatin impregnated	Suits complete aortic arch surgery		
		VASCUTEK^®^ GELWEAVE™ Valsalva	Extended skirt design	Matches aortic root anatomy		
		VASCUTEK^®^ GELWEAVE™ Ante-Flo	Extra branch	Reduces surgery time, Lowers risk of neurological deficits		
		E-VITA™ OPEN PLUS	Extended stented trunk	Suits hybrid surgery procedures	Jotec	
Knitted		INTEGRAD^®^ SILVER	Silver impregnated	Reduces graft infection	Macquet	Aortic, Peripheral
		HEMAGARD^®^ Ultrathin	Collagen coated, Wall thickness = 0.35 mm	Improves healing response, Reduces dilatation, Increases suture strength		Aortic
		HEMASHIELD^®^ GOLD	Collagen coated, Double velour surface	Improves healing response, Reduces dilatation, Increases suture strength		
		VASCUTEK^®^ GELSEAL™	Gelatin impregnated	Improves healing	Terumo	Aortic, Peripheral
		VASCUTEK^®^ GELSOFT™ ERS	Gelatin impregnated, Radially supported	Improves healing and handling		
		FLOWNIT BIOSEAL^®^	Texturised yarn, Collagen impregnated	Low dilatation, Enhances tissue incorporation	Jotec	Aortic

## 5. Conclusions

This paper reviewed the design aspect of medical textiles (woven, knitted, braided, electrospun) intended for vascular implant applications. The three dimensional structure of an arterial wall and its unique mechanical properties (anisotropy, non-linearity, compliance, viscoelasticity) have been widely researched and reported. However, these features remain widely unconsidered while designing synthetic vascular implants. Therefore, the vascular implants used currently in clinics serve the function of a rigid non-distensible conduit but lack the ability to revive lost biomechanical function of the diseased artery. Currently available textile vascular implants are not significantly different from those introduced six decades ago. Their structural geometry is analogous to traditional textile structures rather than to an arterial vessel. This difference is highlighted when late clinical complications arise from behavioural mismatch at the artery-implant anastomosis. These observations ultimately raise the importance of understanding the structure and biomechanics of an artery before adapting a textile structure from its conventional application area to a biological environment consisting of complex structure and functions such as the blood vessel. A synthetic vascular implant with structural characteristics that closely resemble a native artery will present with less complications, which, in turn, translates to a longer-lasting implant.
